# Involvement of the Heme-Oxygenase Pathway in the Antiallodynic and Antihyperalgesic Activity of *Harpagophytum procumbens* in Rats

**DOI:** 10.3390/molecules200916758

**Published:** 2015-09-15

**Authors:** Carmela Parenti, Giuseppina Aricò, Santina Chiechio, Giulia Di Benedetto, Rosalba Parenti, Giovanna M. Scoto

**Affiliations:** 1 Department of Drug Sciences, Pharmacology and Toxicology Section, University of Catania, Viale A. Doria 6, Catania 95125, Italy; E-Mails: cparenti@unict.it (C.P.); chiechio@unict.it (S.C.); scotog@unict.it (G.M.S.); 2 Department of Drug Sciences, Medicinal Chemistry Section, University of Catania, Viale A. Doria 6, Catania 95125, Italy; 3 Department of Biomedical and Biotechnological Sciences, Pharmacology Section, University of Catania, Viale A. Doria 6, Catania 95125, Italy; E-Mail: giuliadibenedetto@libero.it; 4 Department of Biomedical and Biotechnological Sciences, Phisiology Section, University of Catania, Viale A. Doria 6, Catania 95125, Italy; E-Mail:parenti@unict.it

**Keywords:** *Harpagophytum procumbens*, carbon monoxide, allodynia, hyperalgesia, rat, carrageenan

## Abstract

*Harpagophytum procumbens* (*H. procumbens*), also known as Devil’s Claw, has been used to treat a wide range of pathological conditions, including pain, arthritis and inflammation. Inflammatory mediators, released at the site of injury, can sensitize nociceptive terminals and are responsible for allodynia and hyperalgesia. Carbon monoxide (CO), produced in a reaction catalyzed by the enzyme heme oxygenase (HO), may play a role in nociceptive processing and has also been recognized to act as a neurotransmitter or neuromodulator in the nervous system. This study was designed to investigate whether the HO/CO pathway is involved in the analgesic response of *H. procumbens* in carrageenan-induced hyperalgesia in rats. Mechanical allodynia and thermal hyperalgesia were evaluated by using von Frey filaments and the plantar test, respectively. The results of our experiments showed that pretreatment with the HO inhibitor ZnPP IX significantly decreased the antihyperalgesic effect produced by *H. procumbens* (800 mg/kg, i.p.) in carrageenan-injected rats. Consistently, the pretreatment with hemin, a HO-1 substrate, or CORM-3, a CO releasing molecule, before a low dose of *H. procumbens* (300 mg/kg, i.p.) induced a clear antiallodynic response in carrageenan injected rats. These results suggest the involvement of HO-1/CO system in the antiallodynic and antihyperalgesic effect of *H. procumbens* in carrageenan-induced inflammatory pain.

## 1. Introduction

Peripheral tissue injury can result in inflammatory pain, associated with a hypersensitive state due to central and peripheral mechanisms [1]. Pain hypersensitivity is characterized by allodynia (pain produced in response to a non-nociceptive stimulus) and hyperalgesia (increased sensitivity to a painful stimulus) [2,3]. Inflammatory mediators, released in the site of injury, can sensitize nociceptive terminals and are responsible for the pathophysiological changes that participate to the genesis of inflammatory pain [4]. Non steroidal anti-inflammatory drugs are commonly used to reduce inflammation and pain. However, their low efficacy in some forms of chronic pain and the severe adverse effects of these drugs limit their long-term use [5]. Medicinal plants could certainly represent potential agents in therapeutic approaches to the management of pain [6]. Among these, one of the most interesting is *Harpagophytum procumbens* (*H. procumbens*) (Devil’s claw) [7,8,9], a southern African plant and member of the Pedaliaceae family. Its secondary roots provide a traditional drug currently used for a variety of therapeutic effects including pain relief, treatment of arthritis and chronic inflammation. In particular, it has been demonstrated that *H. procumbens* extracts are able to reduce inflammatory pain in Freund’s adjuvant-induced arthritis and in carragenan-induced rat paw oedema [10,11]. However, little is known about the mechanisms underlying the analgesic and anti-inflammatory properties of *H. procumbens*.

Endogenous carbon monoxide (CO) is a gaseous transmitter, involved in nociceptive modulation [12,13]. It arises mainly from the cleavage of heme, a process catalyzed by hemeoxygenase (HO) enzyme of which there are three distinct isoforms, including the constitutive HO-2, HO-3 isoforms, and the inducible HO-1 [14]. The induction of the latter in a variety of cells, such as momocytes/macrophages, neutrophils, endothelial cells, *etc.*, provides negative feedback for the production of inflammatory mediators [15]. The anatomical distribution of HO in nociceptive pathways indicates that the HO/CO system might be involved in pain signaling and transmission [16]. Indeed, treatment with CO—releasing molecules or HO-1 inducers inhibits nociceptive response, supporting the hypothesis that endogenous CO produced by HO plays an antinociceptive role, at least in inflammatory pain [17,18]. HO-1 overexpression results in the inhibition of heme proteins, including cytochrome P-450 isoenzymes and cyclooxygenases (COX) due to diminished heme availability [19]. Moreover, for synthetic or natural compounds, the involvement of the HO-1/CO pathway in their antinflammatory and antinociceptive effects has been highlighted [20,21,22].

The anti-inflammatory effect of *H. procumbens* extract has also been demonstrated in various *in vitro* studies [23,24,25]. In particular, it has been shown that *H. procumbens* elicits a direct inhibitory effect on the COX-2 enzyme. It is also known that COX-2 activity can be affected by the HO-1/CO pathway [19,26]. Thus, the present study was designed to investigate whether the analgesic effect of *H. procumbens* in the carrageenan test was influenced by the integrity of the HO-1/CO pathway. With this aim we evaluated the effect of protoporphyrin IX zinc (II) (ZnPP IX) (a specific HO-1 inhibitor), hemin (a HO-1 inducer) [20] or CORM-3 (a CO-releasing molecule) [27] on the antinociceptive effect of *H. procumbens* in a model of inflammatory pain, such the carrageenan test, in rats.

## 2. Results and Discussion

### 2.1. Mechanical Allodynia

**Figure 1:**
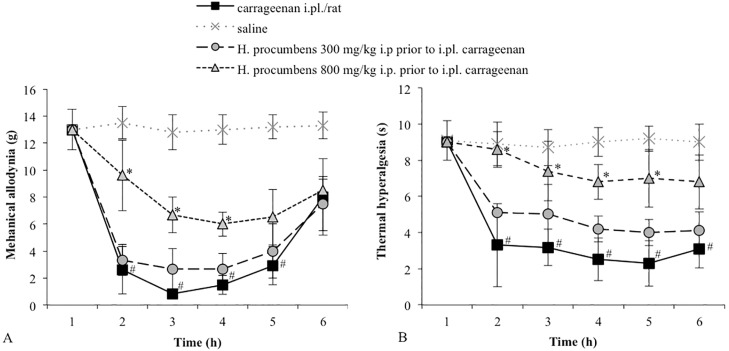
(**A**) Effect of *H. procumbens* (300 and 800 mg/kg i.p.), injected before i.pl. carrageenan (2%/0.1 mL/rat), on mechanical allodynia. Mechanical thresholds were measured with von Frey’s filaments. Results are expressed in grams (g) and represent the means ± SEM from 8 to 10 rats. # *p* < 0.05 *vs.* saline treated-rats; * *p* < 0.05 *vs.* carrageenan injected-rats; (**B**) Effect of *H. procumbens* (300 and 800 mg/kg i.p.), injected before i.pl. carrageenan (2%/0.1 mL/rat), on thermal hyperalgesia. Thermal thresholds were measured with Plantar test. Results are expressed in seconds (s) and represent the means ± SEM from 8 to 10 rats. # *p* < 0.05 *vs.* saline treated-rats; * *p* < 0.05 *vs.* carrageenan injected-rats.

**Figure 2:**
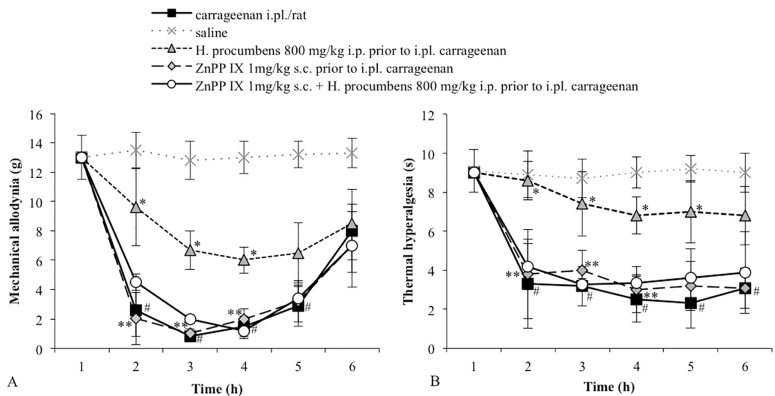
(**A**) Effect of the pretreatment with ZnPP IX (1 mg/kg s.c.) on the antiallodynic effect of *H. procumbens* (800 mg/kg i.p.) in carrageenan-injected rats (2%/0.1 mL/rat). Mechanical thresholds were measured with von Frey’s filaments. Results are expressed in grams (g) and represent the means ± SEM from 8 to 10 rats. * *p* < 0.05 *vs.* carrageenan injected-rats; # *p* < 0.05 *vs.* saline treated-rats; ** *p* < 0.05 *vs. H. procumbens* + carrageenan treated-rats; (**B**) Effect of the pretreatment with ZnPP IX (1 mg/kg s.c.) on the antihyperalgesic (panel B) effect of *H. procumbens* (800 mg/kg i.p.) in carrageenan-injected rats (2%/0.1 mL/rat). Thermal thresholds were measured with Plantar test. Results are expressed in seconds (s) and represent the means ± SEM from 8 to 10 rats. * *p* < 0.05 *vs.* carrageenan injected-rats; # *p* < 0.05 *vs.* saline treated-rats; ** *p* < 0.05 *vs. H. procumbens* + carrageenan treated-rats.

Carrageenan-injected rats exhibited a significant reduction in the mechanical threshold measured with von Frey filaments. This effect lasted for several hours with a peak at 3 h after carrageenan injection (Figure 1A). The control group, receiving i.pl. saline, did not show changes in the basal mechanical threshold. The i.p. administration of *H. procumbens* extract (800 mg/kg) 30 min prior to carrageenan injection significantly reduced carrageenan-induced allodynia. Threshold values significantly differed from the vehicle, from 2 until 4 h (*p* < 0.05). At the lower dose tested (300 mg/kg, i.p.), *H. procumbens* did not significantly modify carrageenan-induced mechanical allodynia (Figure 1A). To investigate the role of HO-1 activity in the antiallodynic effect of the higher dose of *H. procumbens*, animals were pre-treated with the HO-1 inhibitor ZnPP IX (1 mg/kg, s.c.) 30 min before the *H. procumbens* administration. The selected dose of ZnPP IX in our experimental conditions, was not able to modify the mechanical threshold. However, the pretreatment with ZnPP IX completely reverted the antiallodynic effect of *H. procumbens* (Figure 2A).

**Figure 3:**
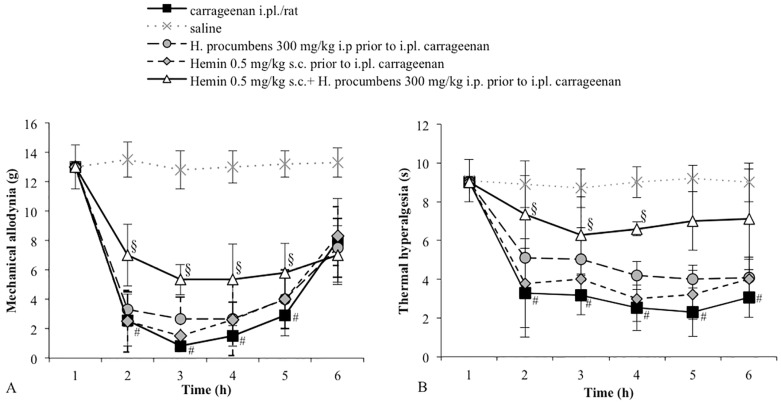
(**A**) Effect of the pretreatment with hemin (0.5 mg/kg s.c.) on the antiallodynic effect of *H. procumbens* (300 mg/kg i.p.) in carrageenan-injected rats (2%/0.1 mL/rat). Mechanical thresholds were measured with von Frey’s filaments. Results are expressed in grams (g) and represent the means ± SEM from 8 to 10 rats. # *p* < 0.05 *vs.* saline treated-rats; § *p* < 0.05 *vs.* carrageenan injected-rats; (**B**) Effect of the pretreatment with hemin (0.5 mg/kg s.c.) on the antihyperalgesic effect of *H. procumbens* (300 mg/kg i.p.) in carrageenan-injected rats (2%/0.1 mL/rat). Thermal thresholds were measured with Plantar test. Results are expressed in seconds (s) and represent the means ± SEM from 8 to 10 rats. # *p* < 0.05 *vs.* saline treated-rats; § *p* < 0.05 *vs.* carrageenan injected-rats.

**Figure 4:**
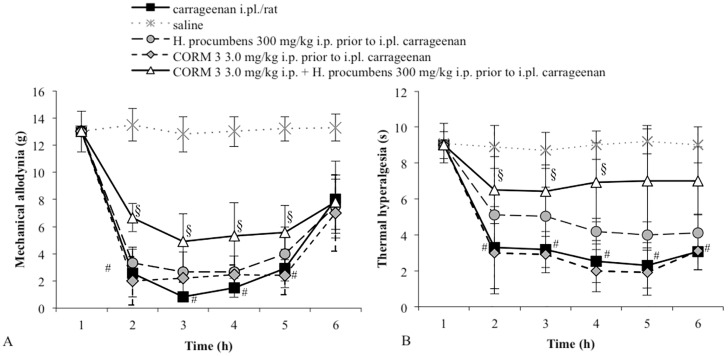
(**A**) Effect of the pretreatment with CORM 3 (3.0 mg/kg i.p.) on the antiallodynic effect of *H. procumbens* (300 mg/kg i.p.) in carrageenan-injected rats (2%/0.1 mL/rat). Mechanical thresholds were measured with von Frey’s filaments. Results are expressed in grams (g) and represent the means ± SEM from 8 to 10 rats. # *p* < 0.05 *vs.* saline treated-rats; § *p* < 0.05 *vs.* carrageenan injected-rats; (**B**) Effect of the pretreatment with CORM 3 (3.0 mg/kg i.p.) on the antihyperalgesic effect of *H. procumbens* (300 mg/kg i.p.) in carrageenan-injected rats (2%/0.1 mL/rat). Thermal thresholds were measured with Plantar test. Results are expressed in seconds (s) and represent the means ± SEM from 8 to 10 rats. # *p* < 0.05 *vs.* saline treated-rats; § *p* < 0.05 *vs.* carrageenan injected-rats.

We then tested whether the pretreatment with hemin, a HO-1 substrate, or CORM-3, a CO releasing molecule (both at doses that per se do not modify the response to a mechanical stimulus), could modify the response of the low dose of *H. procumbens.* Pretreatment with either hemin (0.5 mg /kg, s.c.) (Figure 3A) or CORM-3 (3 mg/kg, i.p.) (Figure 4A) induced a clear antiallodynic response from *H. procumbens* (300 mg/kg, i.p.). All vehicles tested did not significantly differ in carrageenan- or saline-treated groups (data not shown).

### 2.2. Thermal Hyperalgesia

Consistent with our previous study [28], the intraplantar injection of carrageenan produced unilateral progressive behavioral signs of thermal sensitization in the injected hind paw, with a significant reduction of the thermal withdrawal threshold lasting from 2 h to 6 h post-injection with a maximal decrease at 3 h compared to the vehicle-treated paw. Consistently with literature data, *H. procumbens* administration at a dose of 800 mg/kg produced a significant increase in the thermal threshold. On the contrary, *H. procumbens* administration at the lower dose of 300 mg/kg caused only a weak and non-significant increase in thermal threshold in rats (Figure 1B).

To investigate whether the HO-1/CO system is involved in the *H. procumbens* antihyperalgesic effect, we tested the effect of ZnPP IX pretreatment before *H. procumbens* at a dose of 800 mg/kg. Rats were pretreated with the HO-1 inhibitor at a dose of 1 mg/kg, a dose that in our experimental conditions did not modify the response to the thermal stimulus. After 30 min, the animals received a high dose of *H. procumbens* (800 mg/kg, i.p.). The results obtained demonstrated that the pretreatment with ZnPP IX significantly decreased the antihyperalgesic effect induced by *H. procumbens* in carrageenan-injected rats (*p* < 0.05 *vs. H. procumbens*-treated rats). After evaluating the effect of hemin or CORM 3 individually, we chose the respective doses that did not result in a significant shift of thermal threshold. As shown in Figure 3B and Figure 4B, pretreatment with hemin (0.5 mg/kg, s.c.) or with CORM-3 (3 mg/kg, i.p.) before *H. procumbens* (300 mg/kg, i.p.) induced a significant antihyperalgesic response. All vehicles tested did not significantly differ in carrageenan- or saline-treated groups (data not shown).

In this study, we demonstrated the involvement of the HO-1/CO system in *H. procumbens* antiallodynic and antihyperalgesic effects in carrageenan-induced inflammatory pain. In fact, the antinociceptive effect produced by *H. procumbens* was significantly decreased by the administration of the HO-1 inhibitor ZnPP IX, indicating that HO-1 activity participates in *H. procumbens* analgesic effect. Moreover, the pretreatment with the HO-1 inducer compound (hemin) or the CO donor (CORM-3) induced a significant antiallodynic and antihyperalgesic effect in rats treated with a low and ineffective dose of *H. procumbens*.

The main active principle harpagoside, which belongs to the iridoid glycoside family, has been described as being responsible for the antiinflammatory properties of *H. procumbens*, but it is not able, alone, to sufficiently explain the drug’s effects [7,8]. A number of studies [7,8,9,10,29] have investigated the antinociceptive properties of *H. procumbens*, whose efficacy and tolerability was found to be similar, if not superior, to some non-steroidal anti-inflammatory drugs such as acetylsalicylic acid and indomethacin [8]. Pre-treatment of rats with an aqueous extract of *H. procumbens* significantly reduces carrageenan-induced paw oedema and the amount of writhing and stretching induced by 1.2% acetic acid solution [30]. Mahomed and Ojewole [31] have demonstrated that in mice treated with an extract of *H. procumbens*, inflammation induced by fresh albumin is significantly suppressed. Moreover, *H. procumbens* is able to produce significant analgesic activity against chemically and thermally induced nociceptive pain stimuli [31]. Dry extract of *H. procumbens* also exhibits anti-inflammatory and antinociceptive effects in the Freund’s adjuvant-induced arthritis model in rats, being active both in acute and in chronic phase of inflammation [10]. The effect of *H. procumbens* on different inflammatory parameters has also been demonstrated in *in vitro* studies. *H. procumbens* inhibits inflammatory processes by inhibiting cytokine and PGE2 release [25]. An aqueous extract of *H. procumbens* inhibited COX2 induction and PGE2 synthesis in the LPS-treated mouse fibroblast cell line [23] and it has been reported that *H. procumbens* elicits a direct inhibitory effect on the COX-2 enzyme in mouse skin [25] and in human breast epithelial cells [24].

Regulatory interactions between the HO-1 and COX pathways have been reported [13,20]. Cellular heme levels affect COX expression and activity, and HO-1 regulates the expression of vascular COX and the production of the vasoactive prostanoids [19]. Administration of CO releasing molecules, compounds able to reproduce CO biological effects, inhibits nociceptive behavioral response in inflammatory pain models [17,18]. HO-1 inducers or CO donors, such as hemin or CORM-3, have been reported to be analgesic, respectively, at doses of 3 mg/kg s.c. [20] and 5 mg/kg i.p. [27] Conversely, the administration of an HO inhibitor, intrathecally or peripherally injected, determined a significant increase in nociceptive response of rats in formalin test [12,15,16].

In our study we observed that a HO-1 inducer or a CO donor (hemin or CORM-3), at doses that did not modify pain response *per se*, were able to induce an antihyperalgesic effect in rats treated with a subanalgesic dose of *H. procumbens*. Moreover, the antiallodynic and antihyperalgesic effect of *H. procumbens* (800 mg/kg i.p.) was significantly decreased by the pretreatment with ZnPP IX, an HO-1 inhibitor, suggesting that HO-1 activity is involved in the antihyperalgesic effect produced by *H. procumbens* in the carrageenan test. Since the expression of HO-1 is increased at the site of experimental inflammation, which is responsible for peripheral sensitization [12], we hypothesized a peripheral site of action for *H. procumbens*. However, due to the ability of *H. procumbens* to cross the brain blood barrier [32,33], a central site of action cannot be excluded.

It has been demonstrated that treatment with carbon monoxide-releasing molecules or HO-1 inducers enhances the effects of synthetic and natural compounds [22,34,35] and that their ability to reduce inflammatory hypernociception is dependent, at least in part, upon the HO-1 pathway integrity [20,35].

Reactive oxygen species (ROS) can contribute to the tissue injury involved in inflammatory pain [36]. Accordingly, the administration of free radical scavengers reduces the expression of hyperalgesia and allodynia [37]. *H. procumbens* extract dose-dependently increases superoxide dismutase, catalase and glutathione peroxidase activities in brain and reduceslipid peroxidation activity. It has been postulated that the anti-oxidant properties are probably due to flavonoids and phenolic compounds, and that they may be partially responsible for the anti-inflammatory effect of *H. procumbens* extracts [7,38]. A crucial role for antioxidant and tissue protective actions has also emerged for HO-1. Increased HO-1 expression leads to the degradation of heme and accumulation of CO, which may exert tissue protective action. Increased HO-1 expression also increases bilirubin, which has been demonstrated to act as a direct antioxidant, with a consequent reduction of tissue sensitivity to oxidant damage [39,40,41]. Thus increased HO-1 expression and ensuing formation of CO, and also bilirubin, may contribute to antioxidant and anti-inflammatory actions. In conclusion, the present study provides evidence that the antinociceptive effect of *H. procumbens* requires the integrity of the HO-1/CO pathway. Altogether our data indicates that a possible association between *H. procumbens* extracts and CO donors might be of potential interest in the development of clinical agents for the management of inflammatory pain.

## 3. Experimental Section

### 3.1. Animals

Experiments were conducted on male Sprague-Dawley rats (Morini, S. Polo d’Enza, Reggio Emilia, Italy), weighing 180–200 g. The animals were kept at a constant room temperature (25 ± 1 °C) under a 12:12 h light and dark cycle with free access to food and water. Each rat was used for only one experiment. All tests were performed at room temperature (22–24 °C) between 08:00 am and 3:00 pm. Experimental procedures were approved by the Local Ethical Committee and the Institutional Animal Care and Use Committee (IACUC) (M.D. n° 170/2012 issued on 27 August 2012), and all experiments were conducted in accordance with International Guidelines as well as European Communities Council Directive and National Regulations (EEC Council 86/609 and DL 116/92).

### 3.2. Carrageenan-Induced Inflammatory Pain in Rats

Carrageenan was suspended in sterile isotonic (0.9%) saline to a 2% solution and sonicated prior to injection. A volume of 0.1 mL was i.pl. injected into the left hind paw, approximately half way between the toes and heel just proximal to the interdigital pads.

### 3.3. Mechanical Allodynia

The assessment of tactile allodynia consisted of measuring the withdrawal threshold of the hind paw in response to probing with a series of calibrated von Frey’s filaments [3,42]. The rat was placed in a clear plastic testing chamber with a wire mesh bottom and allowed to acclimatize for 20 min. The ventral surface of the hind paw was mechanically stimulated from below with an ascending series of graded von Frey’s filaments with bending forces ranging from 0.02 to 30 g. The paw withdrawal threshold (PWT) was determined by the “up-down” method of sequentially increasing and decreasing the stimulus strength and was expressed as the mean withdrawal threshold.

### 3.4. Thermal Hyperalgesia

Thermal hyperalgesia was quantified using the method described by Hargreaves *et al*. (1988) [3,43]. Briefly, rats were placed in a plexiglass box (17 × 23 × 14 cm) on a glass surface of the apparatus (Plantar test, UgoBasile, Varese, Italy) and a beam of radiant heat was applied through the glass to the plantar surface of the left hind paw. Rats were allowed to habituate to the apparatus until exploratory behaviour was no longer observed. The basal pre-drug latency was established between 8 and 10 s and was calculated as the average of two measurements performed at 5 min intervals with a cut-off latency of 20 s to avoid tissue damage.

### 3.5. Experimental Design

Time course of mechanical allodynia and thermal hyperalgesia evaluation were measured in different animal groups (8–10 rats per group). After the intraplantar (i.pl.) injection of either 0.9% sterile saline (0.1 mL/rat) or carrageenan (2%/0.1 mL/rat) rats were tested for 6 h post-injection; *H. procumbens* extract (300 and 800 mg/kg i.p.) or vehicle were administered 30 min prior to i.pl. carrageenan (2%/0.1 mL/rat); protoporphyrin IX zinc (II) (ZnPP IX) (1 mg/kg s.c.) or vehicle, hemin (0.5 mg/kg s.c.) or vehicle and carbon monoxide releasing molecule 3 (CORM-3) (3 mg/kg i.p) or vehicle were administered either alone (60 min prior to i.pl. carrageenan), or 30 min before *H. procumbens* extract.

### 3.6. Drugs

Carrageenan (mixture of κ and λ carrageenan), protoporphyrin IX zinc(II) (ZnPP IX; a specific HO-1 inhibitor), hemin (an HO-1 substrate), Carbon monoxide releasing molecule 3 (CORM-3; a CO donor) was supplied by Sigma-Aldrich (Milan, Italy). *H. procumbens* standardized freeze-dried extract was kindly donated by Aboca (San Sepolcro, Italy), batch number 10J1398. Carrageenan, CORM-3 and *H. procumbens* extract were dissolved in 0.9% sterile saline; ZnPP IX was dissolved in 50 mM Na2CO3; Hemin was dissolved in 1 mM NaOH.

#### Plant Extract

The dried root from Namibia (Africa), was extracted with ethanol 70°. At the end of extraction, ethanol was evaporated under vacuum and the resulting mixture was freeze-dried yielding the correspondent extract (yield: 28.5%, DER 3.5:1). The sample of *H. procumbens* freeze-dried extract is standardized at 2% of harpagoside content. *H. procumbens* standardized freeze-dried extract (0.25 g) was treated with 50 mL of the solvent mixture Methanol/Water 80/20 for 30 min, at room temperature under ultrasonic treatment. The resulting mixture was centrifuged, filtered into a volumetric flask, and brought to the final volume of 50 mL with the same solvent mixture. All of the extracts were filtered through 0.45 mm Cellulose acetate syringe filter before HPLC analysis.

Harpagoside standard (purity 99.9%, HPLC grade) was purchased from Phytolab. A 1 mg/mL stock solution was diluted to obtain samples with harpagoside concentration ranging from 0.025 to 0.1 mg/mL. Injections of each standard concentration were made in tripled. The calibration curve was obtained by plotting the mean peak areas *vs.* the corresponding concentrations.

### 3.7. Statistical Analysis

Data are expressed as the mean ± SEM. Intergroup comparisons were assessed by analysis of variance (ANOVA), followed by *Post-hoc* test (Bonferroni). A *p* value less than 0.05 (*p* < 0.05) was considered statistically significant.
